# Preemptive treatment in the acute and early subacute phase of uncomplicated type B aortic dissections with poor prognosis factors

**DOI:** 10.3389/fcvm.2024.1362576

**Published:** 2024-04-26

**Authors:** Charlotte Sachs, Fabien Vecchini, Marie Corniquet, Michel Bartoli, Pierre-Antoine Barral, Mariangela De Masi, Virgile Omnes, Philippe Piquet, Jean-Marc Alsac, Marine Gaudry

**Affiliations:** ^1^ Department of Vascular Surgery, APHM, Timone Hospital, Marseille, France; ^2^ Aortic Center, APHM, Timone Hospital, Marseille, France; ^3^ Department of Vascular Surgery, APHP, Georges Pompidou European Hospital, Paris, France; ^4^ Department of Radiology, APHM, Timone Hospital, Marseille, France

**Keywords:** preemptive endovascular treatment, uncomplicated type B aortic dissection, risk factors, aneurysmal evolution, optimal medical treatment, comparative study

## Abstract

**Objective:**

Due to its favorable outcome regarding late morbidity and mortality, thoracic endovascular repair (TEVAR) is becoming more popular for uncomplicated type B aortic dissection (TBAD). This study aimed to compare preemptive endovascular treatment and optimal medical treatment (OMT) and OMT alone in patients presenting uncomplicated TBAD with predictors of aortic progression.

**Design:**

Retrospective multicenter study

**Methods:**

We analyzed patients with uncomplicated TBAD and risk factors of progression in two French academic centers. Aortic events [defined as aortic-related (re)intervention or aortic-related death after initial hospitalization], postoperative complications, non-aortic events, and radiologic aortic progression and remodeling were recorded and analyzed. Analysis was performed on an intention-to-treat basis.

**Results:**

Between 2011 and 2021, preemptive endovascular procedures at the acute and early subacute phase (<30 days) were performed on 24 patients (group 1) and OMT alone on 26 patients (group 2). With a mean follow-up of 38.08 ± 24.53 months, aortic events occurred in 20.83% of patients from group 1 and 61.54% of patients from group 2 (*p* < .001). No patient presented aortic-related death during follow-up. There were no differences in postoperative events (*p* = 1.00) and non-aortic events (*p* = 1.00). OMT patients had significantly more aneurysmal progression of the thoracic aorta (*p* < .001) and maximal aortic diameter (*p* < .001). Aortic remodeling was found in 91.67% of patients in group 1 and 42.31% of patients in group 2 (*p* < .001). A subgroup analysis of patients in group 1 showed that patients treated with preemptive TEVAR and STABILISE had reduced maximum aortic diameters at the 1-year (*p* = .010) and last follow-up (*p* = .030) compared to those in patients treated with preemptive TEVAR alone.

**Conclusion:**

Preemptive treatment of uncomplicated TBAD with risk factors of progression reduces the risk of long-term aortic events. Over 60% of medically treated patients will require intervention during follow-up, with no benefit in terms of postoperative events. Even after surgical treatment, patients in the OMT group had significantly more aneurysmal progression, along with poorer aortic remodeling.

## Introduction

With a serious and fatal prognosis, type B aortic dissection (TBAD) is a rare condition with an incidence of 3.5/100,000 persons per year (1, 2). Optimal medical treatment (OMT) is mandatory in all TBAD, and thoracic endovascular repair (TEVAR) in the acute phase is advised for complicated TBAD (3–7).

The INSTEAD study (4) is the only randomized trial to have shown the long-term benefits of TEVAR compared to OMT alone in uncomplicated subacute and chronic TBAD with regard to the prevention of aortic aneurysm progression and aortic-specific mortality (4, 8). In view of these results, preemptive TEVAR may be considered in high-risk patients with uncomplicated TBAD (grade IIB recommendation) (3, 5–7, 9).

Several risk factors are known to promote aneurysmal progression of the descending aorta after TBAD: young age, aortic diameter > 40 mm, patent false lumen (FL), ratio of true lumen (TL) to FL, and primary entry tear (PET)  > 10 mm (10–12). All these factors can be used to identify patients who may benefit from preemptive endovascular intervention.

Acute dissection is more likely to be associated with life-threatening complications than either subacute or chronic dissection (13). Recent series have suggested that TEVAR for subacute or chronic TBAD has a lower mortality and complication rate than for acute TBAD (14, 15). The chronicity of the dissection also has relevance with regard to aortic remodeling after TEVAR, which is significantly greater in patients with acute or subacute dissection. TEVAR of acute or subacute dissection is associated with rapid expansion of the TL and collapse of the FL. In contrast, TEVAR for chronic dissection can induce FL thrombosis in the treated segment without a change in aortic diameter and with a patent FL on the thoracoabdominal (TA) aorta (16).

This study aimed to evaluate the mid and long-term results of preemptive endovascular treatment and OMT in the acute and early subacute phase (<30 days) as compared to OMT alone for the treatment of uncomplicated TBAD with predictors of aortic growth.

## Methods

### Population

All patients included in this study were informed about the use of their data for clinical research. The institutional review board approved the project (No. MCBFBR).

We performed a retrospective study in two French university hospitals. The inclusion criteria were patients admitted between August 2011 and December 2021, who were under 70 years of age, presenting uncomplicated TBAD [defined as any TBAD presenting without rupture, malperfusion; renal, mesenteric, limbic, medullar, and uncontrolled hypertension (HTA); or uncontrolled pain], with predictors of aortic progression.

The patients presenting at least one risk factor were included. The risk factors were defined as follows:

• Maximum aortic diameter, >40 mm
• FL diameter, >20 mm
• PET, >10 mm

The patients were excluded if one of the following criteria was found:

• Residual AD after type A repair
• Non-A/non-B dissection
• Intramural hematoma, penetrating aortic ulcer, or traumatic isthmus rupture
• Complicated TBAD during initial hospitalization
• Patients aged >70 years old
• Patients with maximum aortic diameter of >55 mm
• Patients with unavailable initial scanner or without follow-up at >1 year
• Patients without risk factors

The patients were divided into two groups: group 1 received preemptive endovascular treatment and OMT at the acute or early subacute phase (<30 days), and group 2 received OMT alone.

### Patient treatment

#### OMT

All patients were admitted to the intensive care unit (ICU) for at least 48 h. HTA was controlled with intravenous beta-blockers and calcium-channel blockers with a goal of systolic blood pressure of <120 mmHg. Chest pain was relieved with non-narcotic and narcotic analgesics. Each patient had a follow-up CT scan at 48 h before transfer to the regular ward.

#### Endovascular treatment

All patients in group 1 were treated within 30 days post-AD. Since 2014, TEVAR has been performed in a multimodal angiographic suite. The stent was deployed using the standard technique. Four types of prosthesis were implanted: C-TAG (W. L. Gore & Associates Inc., Flagstaff, Arizona), Valiant Navion (Medtronic, Santa Rosa, California), RELAY NBS (Terumo Aortic), and Zenith (Cook Medical, Bloomington, Indiana). The choice of the stent graft and the debranching of the left subclavian artery (LSA) were left to the discretion of the surgeon. The choice of proximal implantation site was based on the location of the PET. The stent size was determined by measurements at the proximal and distal implantation sites in an orthogonal plane using a centerline reconstruction on the preoperative CT scan. Proximal and distal oversizing of 10% was performed. Cerebrospinal fluid drainage (CFD) was performed when there was extensive coverage of the thoracic aorta with a stent graft (>250 mm) in the absence of contraindications.

Depending on the choice of the surgeon, some patients benefited from the Stent-Assisted Balloon-Induced Intimal Disruption and Relamination of Aortic Dissection (STABILISE) technique (17, 18).

### Endpoints

The primary endpoint was the aortic event rate defined as any of the following events occurring after initial hospitalization:

• Aortic-related (re)interventions (patients in group 1 requiring reintervention after initial surgery or patients in group 2 requiring an intervention for any of these events)
  • Aortic rupture
  • Malperfusion syndrome (renal insufficiency, mesenteric, limb, or medullar ischemia)
  • Aortic aneurysm evolution (>10 mm/year or a diameter of >55 mm)
  • Retrograde type A AD
• Aortic-related death during follow-up

**Figure 1:**
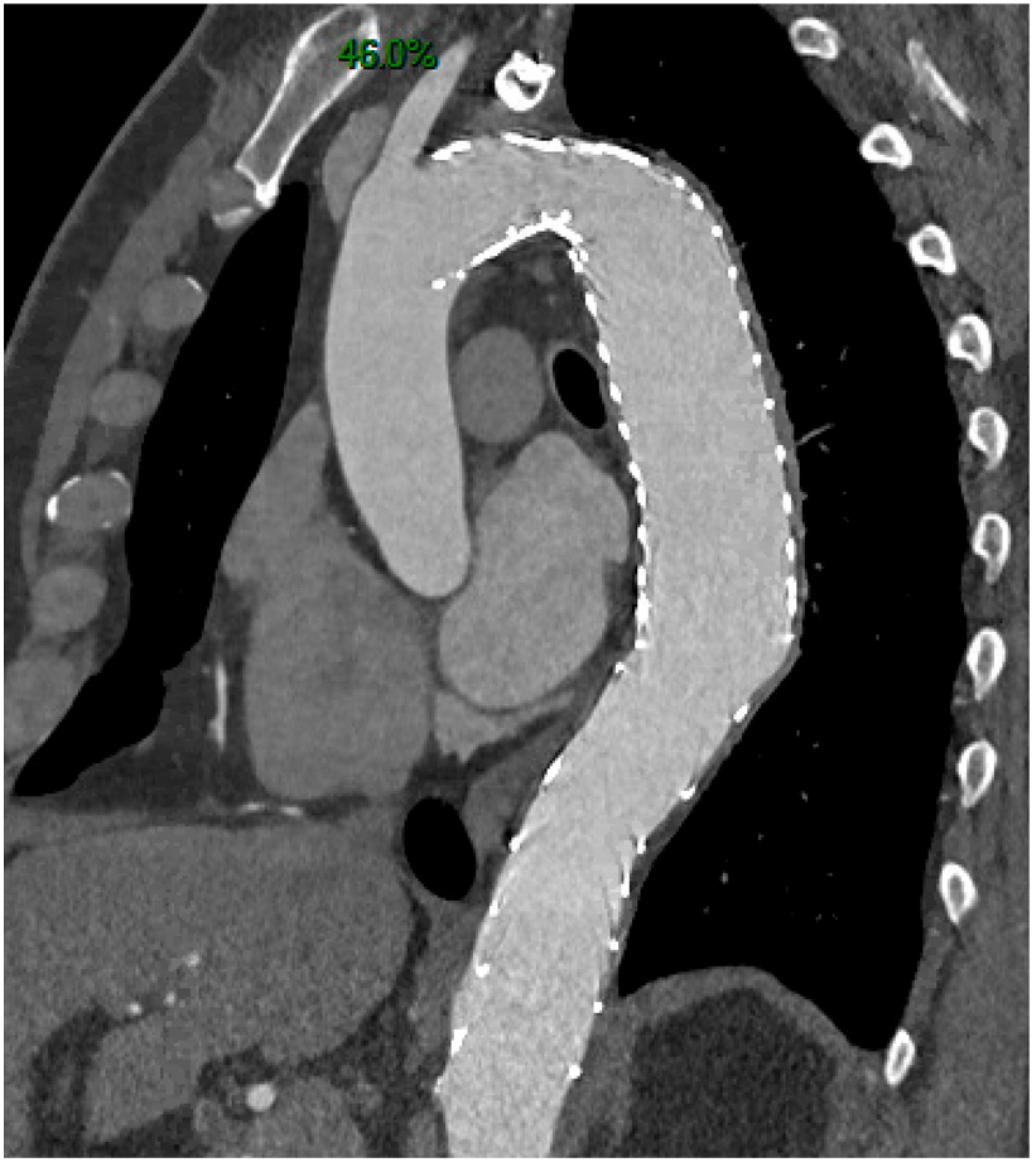
Complete aortic remodeling with false lumen shrinkage.

The secondary endpoints were as follows:

• Postoperative events in both groups (30 days following preemptive surgery in group 1 or surgery performed in case of complications in group 2)
• Non-aortic events occurring after initial hospitalization
• Radiologic aortic analysis consisting of aneurysmal progression of aortic diameters (defined as a 5 mm increase in the aortic diameter within 6 months or an aortic diameter reaching 50 mm during follow-up) and aortic remodeling [FL thrombosis and reapposition of the intimal flap (Figure 1)].

### Radiological analysis

Scan image analysis and measurements were performed using three-dimensional imaging software (Osiris software, Geneva, Switzerland). Diameter measurements were performed on the perpendicular axis according to the centerline using a semiautomated centerline algorithm on the initial CT scan and at the 6-month, 12-month, and last follow-up CT scan available at different levels: at the level of the descending thoracic aorta, the TA aorta, and the abdominal aorta. The maximum aortic diameter of the thoracic aorta was measured for each patient.

### Statistical analysis

Data are presented as the mean ± standard deviation for continuous variables and as counts (%) for categorical data. For categorical variables, the relationship between variables was studied using the *χ*^2^ test or Fisher's exact test as appropriate. The Wilcoxon and Student’s tests were used to analyze continuous variables. The normality of the distribution was assessed with the Shapiro–Wilk test. Intervention-free survival was estimated with a Kaplan–Meier curve. The subgroup analyses were performed using a Kruskal–Wallis test. All statistical analyses were performed using R software version 4.2.2 (R Foundation for Statistical Analysis, Vienna, Austria).

## Results

### Baseline characteristics

Between August 2011 and December 2021, 50 patients from two French hospitals were included: 24 had preemptive endovascular treatment in addition to OMT (group 1) whereas 26 were treated with OMT alone (group 2). The baseline characteristics and comorbidity are presented in Table 1.

**Table 1:** Baseline characteristics of patients in group 1 (preemptive endovascular treatment in addition to OMT) and group 2 (OMT alone).

	Group 1	Group 2	Total	*p*-value
	*n* = 24	*n* = 26	*n* = 50	
Male sex, *n* (%)	23 (95.83)	18 (69.23)	41 (82.00)	.040
Age, mean (SD)	61 (13.85)	58.15 (8.81)	59.52 (11.48)	.40
Hypertension, *n* (%)	21 (87.50)	23 (88.46)	44 (88.00)	1.00
Diabetes, *n* (%)	2 (8.33)	2 (7.69)	4 (8.00)	1.00
Hypercholesterolemia, *n* (%)	3 (12.50)	8 (30.77)	11 (22.00)	.22
Obesity, *n* (%)	3 (12.50)	4 (15.38)	7 (14.00)	1.00
Smoker, *n* (%)	9 (37.50)	17 (65.38)	26 (52.00)	.090
Connective tissue disorder, *n* (%)	5 (20.83)	3 (11.54)	8 (16.00)	.61
Family history of AD, *n* (%)	0 (0.00)	1 (3.85)	1 (2.00)	1.00
ASA score, *n* (%)				.41
ASA 2	6 (27.27)	4 (16.00)	10 (21.28)	
ASA 3	11 (50.00)	11 (44.00)	22 (46.81)	
ASA 4	0 (0.00)	2 (8.00)	2 (4.26)	
Comorbidities: *n* (%)				
Myocardial infarction	2 (8.33)	1 (3.85)	3 (6.00)	.94
Chronic renal failure	0 (0.00)	2 (7.69)	2 (4.00)	.51
Atrial fibrillation	3 (12.50)	1 (3.85)	4 (8.00)	.55
Stroke	0 (0.00)	2 (7.69)	2 (4.00)	.51
Cancer	2 (8.33)	0 (0)	2 (4.00)	.51
Mean follow-up, months (SD)	41.54 (22.04)	34.88 (26.66)	38.08 (24.53)	.34
Radiological features, mean (SD)				
Primary entry tear	15.93 (7.34)	13.39 (5.31)	14.35 (6.18)	.27
Maximum aortic diameter	43.08 (5.74)	39.19 (5.01)	41.06 (5.67)	.040
Maximum FL diameter	24.38 (7.20)	24.42 (6.20)	24.40 (6.63)	.96

AD, aortic dissection; FL, false lumen.

The patients in group 1 had a significantly larger maximum aortic diameter than that in patients in group 2: 43.08 mm vs. 39.19 mm, respectively (*p* = .040). The mean follow-up time was 38.08 months: 41.54 months in group 1 vs. 34.88 months in group 2 (*p* = .34).

In group 1, 5 patients had a TEVAR procedure (20.83%), 19 had a TEVAR and STABILISE procedure (79.17%), 9 (37.50%) had CFD, 7 (29.17%) had LSA coverage, and none presented spinal cord ischemia. The procedure details are presented in Table 2.

**Table 2:** Procedure details.

	Group 1	Group 2
	*n* = 24	*n* = 26
Proximal neck management; *n*		
Coverage of the left subclavian artery	7	0
Left subclavian bypass	3	1
Left subclavian debranching	3	5
Intra thoracic supra-aortic branch debranching	1	2
Devices, *n*		
Gore	19	6
Medtronic	0	2
Terumo Aortic	4	1
Cook	1	1
Branched arch endograft	0	3
CFD, *n*	9	3

CFD, cerebrospinal fluid drainage.

### Aortic events

Aortic events occurred in 5 patients in group 1 (20.83%) and 16 patients in group 2 (61.54%) (*p* < .001). The mean time to (re)intervention was 13.4 (±14.67) and 8.37 (±11.26) months in groups 1 and 2, respectively (*p* = .90) (Table 3).

**Table 3:** Aortic events in group 1 (preemptive endovascular treatment in addition to OMT) and group 2 (OMT alone).

	Group 1	Group 2	*p-*value
	*n* = 24	*n* = 26	
Aortic events, *n* (%)	5 (20.83)	16 (61.53)	<.001
Aortic-related deaths, *n* (%)	0 (0.00)	0 (0.00)	1.00
Rupture, *n* (%)	0 (0.00)	0 (0.00)	1.00
Mean delay to intervention, months (SD)	13.4 (14.67)	8.37 (11.26*)*	.90
(Re)intervention for aortic progression, *n* (%)	4 (16.66)	15 (57.69)	<.001
TEVAR	1 (4.17)	4 (15.38)	.35
TEVAR–STABILISE	1 (4.17)	7 (26.92)	.050
TEVAR angioplasty	1 (4.17)	0 (0.00)	.48
Open repair of TAA	1 (4.17)	1 (3.85)	1.00
Branched arch endograft	0 (0.00)	3 (11.54)	.24
(Re)intervention for malperfusion, *n* (%)	1 (4.17)	1 (3.85)	1.00
(Re)intervention for retrograde type A AD, *n* (%)	0 (0.00)	2 (7.69)	.49

AD, aortic dissection.

We observed no aortic-related death and no aortic rupture in both groups (*p* = 1.00).

The main aortic event was (re)intervention for aneurysmal progression: 4 patients (16.66%) in group 1 and 15 patients (57.69%) in group 2 (*p* < .001):

In group 1, one patient (4.17%) was managed by extension by TEVAR alone and one (4.17%) by open TA aneurysm repair. Two patients progressed due to type 1B endoleak: 1 patient (4.17%) was managed by extension of TEVAR–STABILISE, and the other required distal TEVAR angioplasty. Out of these four patients, three had been treated with preemptive TEVAR alone.

In group 2, seven patients (26.92%) were operated by TEVAR–STABILISE, four patients (15.38%) by TEVAR, three patients (11.54%) by branched arch endograft, and one patient (3.85%) by open TA aneurysm repair. Three of these patients had CFD due to extensive coverage. No LSA was covered in this group (Table 2).

One patient in group 1 (4.17%) and another in group 2 (3.95%) had a malperfusion requiring intervention (*p* = 1.00):

One patient in both groups had lower limb ischemia, managed by extension of STABILISE and iliac kissing in group 1 and TEVAR associated with stenting of the right primary iliac artery in group 2.

Two patients in group 2 (7.69%) and none in group 1 had retrograde type A AD after TEVAR management (both performed for aneurysmal evolution): in one case, retrograde type A AD occurred 8 days postoperative and 4 years after the procedure in the other case (*p* = .49).

**Figure 2:**
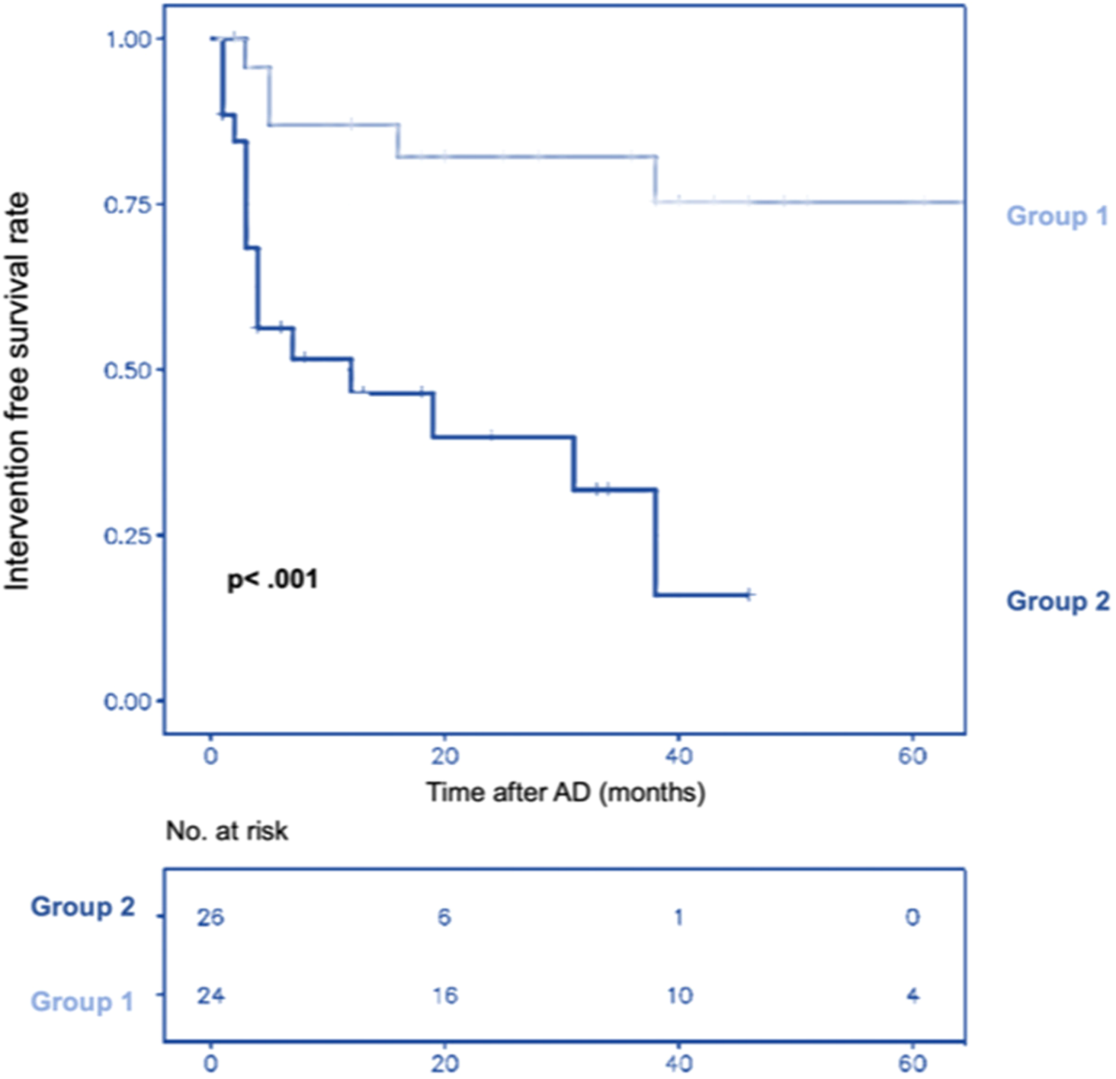
Cumulative Kaplan–Meier estimate of patient survival without intervention over time after aortic dissection (AD) in group 1 (preemptive endovascular treatment in addition to OMT) and group 2 (OMT alone).

The intervention-free survival rate estimated by the Kaplan–Meier curve at 1–3 years was 88%, 81%, and 81% for group 1% vs. 52%, 40%, and 31% for group 2 [HR = 0.18, CI (0.06–0.51)] (*p* < .001) (Figure 2).

### Postoperative events

We observed two postoperative events in group 1 (8.33%) and 3 (11.54%) in group 2 (*p* = 1.00). No postoperative deaths occurred (*p* = 1.00) (Table 4).

**Table 4:** Postoperative events in group 1 (preemptive endovascular treatment in addition to OMT) and group 2 (OMT alone).

Colonne1	Group 1	Group 2	*p-*value
	*n* = 24	*n* = 26	
Postoperative events: *n* (%)	2 (8.33)	3 (11.54)	1.00
Death <30 days	0 (0.00)	0 (0.00)	1.00
Acute renal failure	1 (4.17)	1 (3.85)	1.00
Stroke	1 (4.17)	0 (0.00)	.48
Paraplegia	0 (0.00)	2 (7.69)	.49

In group 1, one patient (4.17%) had an acute renal failure (ARF) and bleeding due to a perioperative renal artery wound, requiring embolization and transient dialysis for 4 days, and one patient (4.17%) had a stroke with sequelae hemiparesis. The patients had been treated preemptively at 4 and 6 days, respectively, after TBAD.

In group 2, one patient (3.85%) had ARF and bleeding after TEVAR (for aneurysmal progression), requiring embolization of his inferior polar renal artery.

Two patients (7.69%) had transient paraplegia after TEVAR–STABILISE and a branched arch endograft (both for aneurysmal progression), with complete recovery after CFD.

### Non-aortic events

We observed three non-aortic events in group 1 (12.5%) and two in group 2 (11.54%) (*p* = 1.00). There was one death in each group, all due to neoplastic disease (*p* = 1.00).

In group 1, one patient (4.17%) had iliac stent thrombosis treated by endovascular recanalization. Another patient (4.17%) developed ARF at 7 months after TEVAR–STABILISE due to renal stent thrombosis, managed by thromboaspiration and angioplasty.

In group 2, two patients (7.69%) developed ARF. One non-operated patient had ARF managed by modification of his antihypertensive treatment. One patient developed renal malperfusion after TEVAR STABILISE (aneurysmal progression), requiring renal stenting.

### Radiological analysis

**Figure 3:**
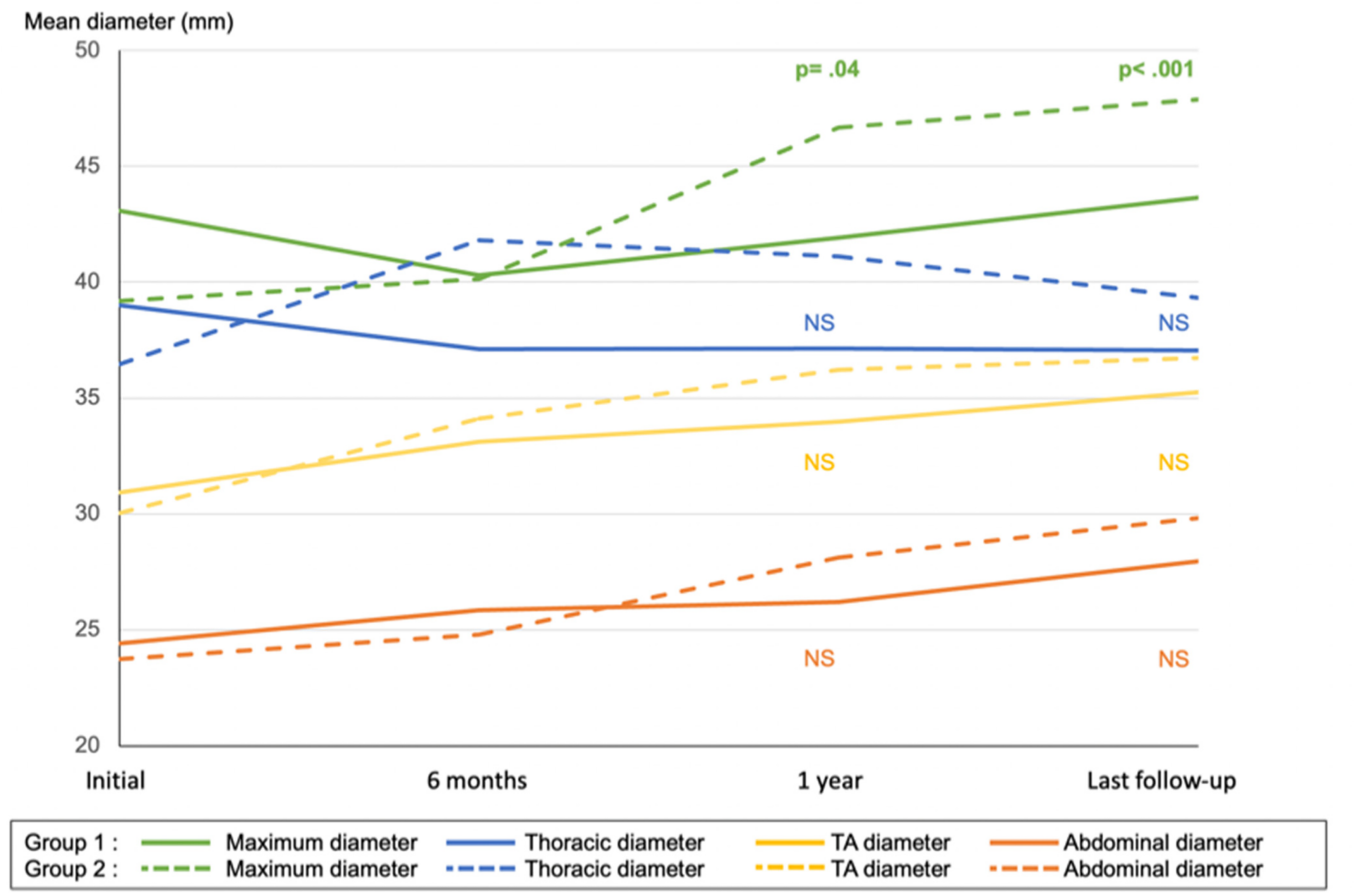
Mean aortic diameter of maximum, thoracic, thoracoabdominal (TA), and abdominal aorta in group 1 (preemptive endovascular treatment in addition to OMT) and group 2 (OMT alone). NS, non-significant.

Preemptive endovascular treatment was associated with a significant decrease in maximum aortic diameter at the 1-year (*p* = .04) and last follow-up (*p* < .001) compared to OMT alone (Figure 3).

There were significantly more patients with aneurysmal progression in group 2 (Table 5).

**Table 5:** The number of patients with aneurysmal progression of aortic diameters (defined as a 5 mm progression within 6 months or an aortic diameter reaching 50 mm) and patients presenting with complete aortic remodeling at the 6-month, 1-year, and last follow-up in group 1 (preemptive endovascular treatment in addition to OMT) and group 2 (OMT alone).

Colonne1	Group 1	Group 2	*p*-value
	*n* = 24	*n* = 26	
6 months			
Thoracic aorta, *n* (%)	2 (8.33)	13 (50.00)	<.001
Thoracoabdominal aorta, *n* (%)	6 (25.00)	7 (26.92)	1.00
Abdominal aorta, *n* (%)	4 (16.67)	3 (11.54%)	.70
Maximum aortic diameter, *n* (%)	3 (12.50)	12 (46.15)	.010
Diameter >50 mm, *n* (%)	2 (8.33)	5 (19.23)	.42
Aortic remodeling, *n* (%)	18 (78.26)	2 (7.69)	<.001
1 year			
Thoracic aorta, *n* (%)	3 (12.50)	13 (50.00)	.01
Thoracoabdominal aorta, *n* (%)	9 (37.50)	10 (38.46)	1.00
Abdominal aorta, *n* (%)	6 (25.00)	5 (19.23)	.74
Maximum aortic diameter, *n* (%)	2 (8.33)	16 (61.54)	<.001
Diameter >50 mm, *n* (%)	2 (8.33)	8 (30.77)	.080
Aortic remodeling, *n* (%)	22 (91.67)	8 (32.00)	<.001
Last follow-up			
Thoracic aorta, *n* (%)	2 (8.33)	14 (53.85)	<.001
Thoracoabdominal aorta, *n* (%)	10 (41.67)	10 (38.46)	1.00
Abdominal aorta, *n* (%)	8 (33.33)	6 (23.08)	.53
Maximum aortic diameter, *n* (%)	3 (12.50)	20 (76.92)	<.001
Diameter >50 mm, *n* (%)	2 (8.33)	8 (30.77)	.080
Aortic remodeling, *n* (%)	22 (91.67)	11 (42.31)	<.001

On the last follow-up CT scan, 2 (8.33%) and 14 (53.85%) patients had aneurysmal progression of the thoracic aorta in groups 1 and 2, respectively (*p* < .001).

Three patients in group 1 (12.50%) and 20 (76.92%) in group 2 had aneurysmal progression of the maximum aortic diameter (*p* < .001).

Two patients (8.33%) in group 1 and eight patients (30.77%) in group 2 had a diameter of >50 mm at the end of follow-up (*p* = .080).

There was no difference in the aneurysmal progression of the TA aorta: 10 (41.67%) patients in group 1 and 10 (38.46%) patients in group 2 had an aneurysmal evolution at the last follow-up (*p* = 1.00) (Table 5).

**Figure 4:**
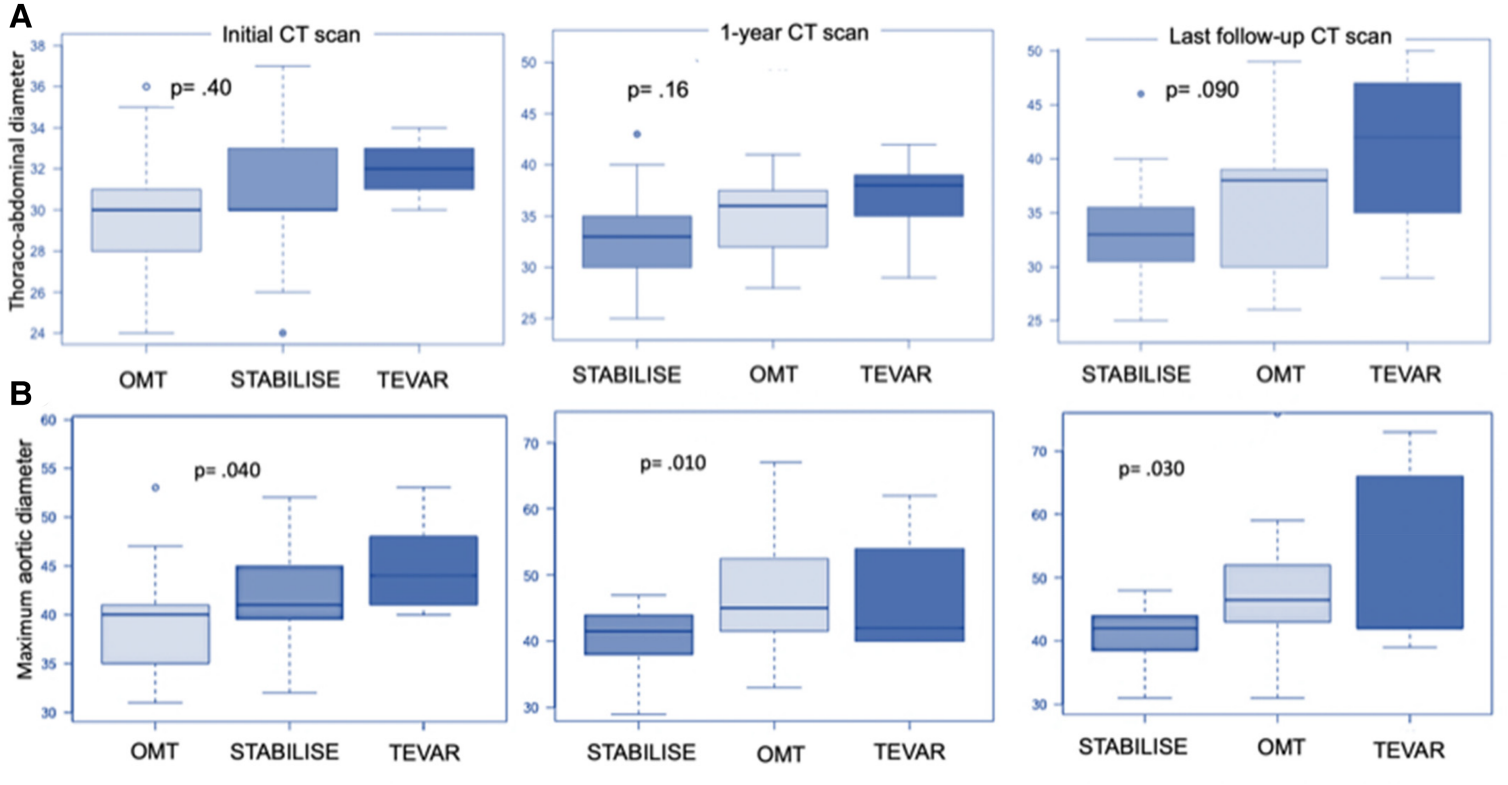
Boxplot showing thoracoabdominal diameter (**A**) and maximum aortic diameter (**B**) in patients treated by OMT alone (group 2) and the subgroup analysis of patients in group 1 treated by preemptive TEVAR or by preemptive TEVAR and STABILISE. Initially, thoracoabdominal diameters were 30.0 mm ± 3.2 mm, 30.6 mm ± 3.2 mm, and 32.0 mm ± 1.6 mm for patients treated with OMT, TEVAR and STABILISE, and TEVAR, respectively (*p* = .40). At 1-year follow-up, the diameters were 33.2 mm ± 14.2 mm, 36.2 mm ± 15.8 mm, and 36.6 mm ± 14.9 mm for patients treated with TEVAR and STABILISE, OMT, and TEVAR, respectively (*p* = .16). At the last follow-up, the diameters were 33.5 mm ± 14.9 mm, 36.7 mm ± 16.9 mm, and 40. mm ± 18.6 mm for patients treated with TEVAR and STABILISE, OMT, and TEVAR, respectively (*p* = .090). (**A**) Initially, the maximum aortic diameters were 39.4 mm ± 4.9 mm, 42.5 mm ± 5.8 mm, and 45.2 mm ± 5.3 mm for patients treated with OMT, TEVAR and STABILISE, and TEVAR, respectively (*p* = .040). At 1-year follow-up, the diameters were 40.3 mm ± 4.6 mm, 46.6 mm ± 8.1 mm, and 47.6 mm ± 9.9 mm for patients treated with TEVAR and STABILISE, OMT, and TEVAR, respectively (*p* = .010). At the last follow-up, the diameters were 40.7 mm ± 4.7 mm, 47.8 mm ± 10.1 mm, and 52.4 mm ± 15.8 mm for patients treated with TEVAR and STABILISE, OMT, and TEVAR, respectively (*p* = .030) (**B**).

However, the subgroup analysis of patients in group 1 showed that patients treated with preemptive TEVAR alone and OMT-treated patients were more likely to progress on the TA aorta than patients treated by TEVAR–STABILISE (*p* = .090) (Figure 4A).

The subgroup analysis of patients in group 1 showed that patients treated with preemptive TEVAR and STABILISE had reduced maximum aortic diameters at the 1-year (*p* = .010) and last follow-up compared to patients treated with preemptive TEVAR alone (*p* = .030) (Figure 4B).

**Figure 5:**
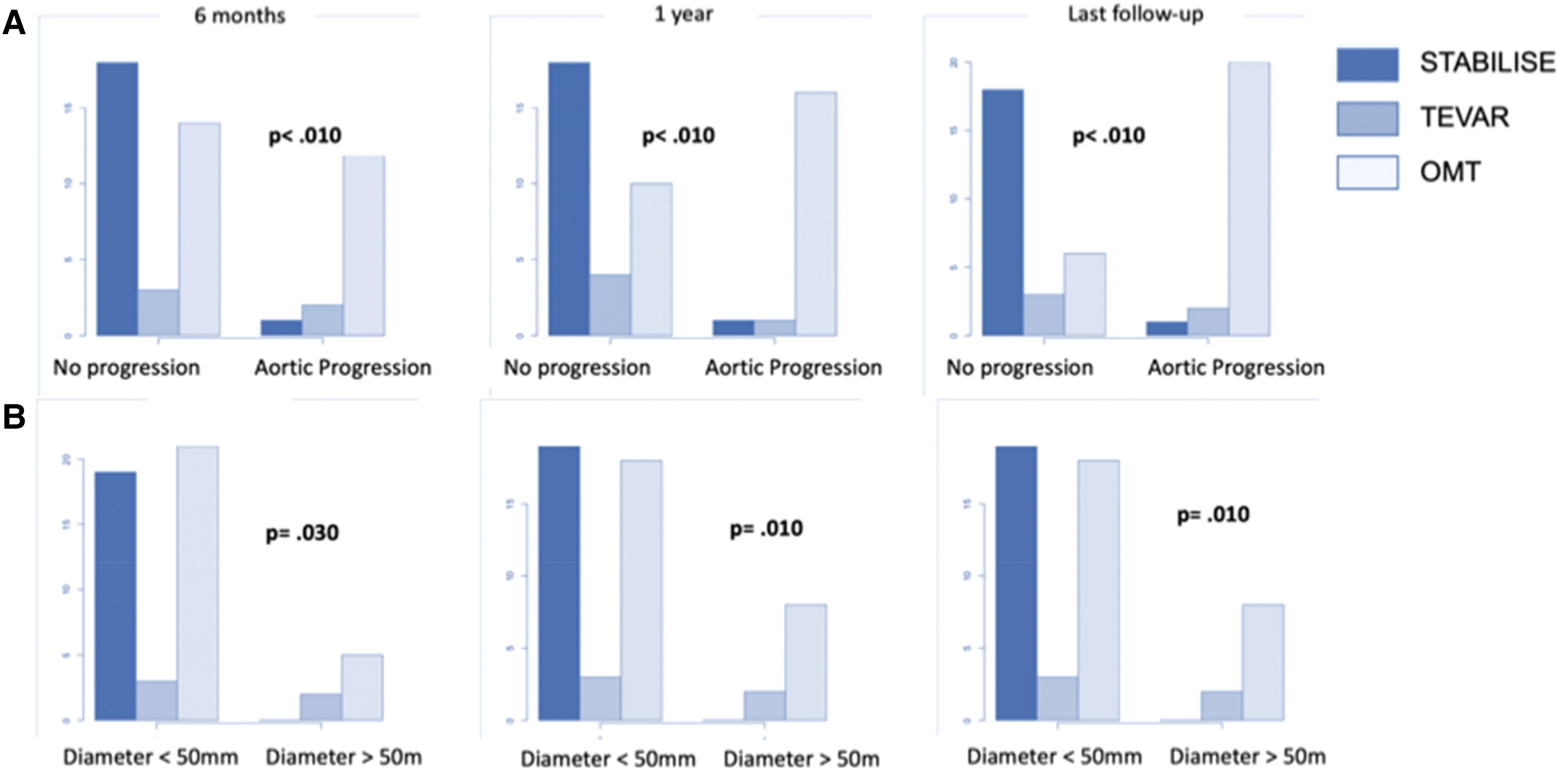
The number of patients with aneurysmal progression of the maximum thoracic diameter (**A**) or reaching a diameter >50 mm (**B**) in patients treated by OMT alone (group 2) and the subgroup analysis of patients in group 1 treated by preemptive TEVAR or by preemptive TEVAR and STABILISE.

The patients treated with preemptive TEVAR and STABILISE were less likely to present aortic progression of maximum aortic diameter compared to the patients treated with preemptive TEVAR and OMT alone (*p* < .010) (Figure 5A).

No patients treated with preemptive TEVAR and STABILISE had any aortic diameter reaching 50 mm compared to patients treated with preemptive TEVAR and OMT alone (*p* < .010) (Figure 5B).

Complete aortic remodeling was obtained in 22 patients (91.67%) in group 1 and 11 patients (42.31%) in group 2 (*p* < .001) (Table 5).

**Figure 6:**
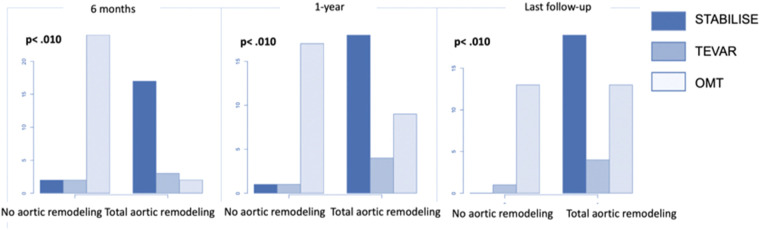
The number of patients with total aortic remodeling in patients treated by OMT alone (group 2) and the subgroup analysis of patients in group 1 treated by preemptive TEVAR or by preemptive TEVAR and STABILISE.

The subgroup analysis showed that patients in group 1 treated with preemptive TEVAR–STABILISE were more likely to have aortic remodeling than patients treated with preemptive TEVAR alone and OMT alone (*p* < .001) (Figure 6).

## Discussion

In this study, we showed that preemptive treatment (<30 days) of uncomplicated TBAD with predictors of aortic growth is associated with a reduced risk of reintervention, aneurysmal progression, and increased aortic remodeling.

Indeed, 20.83% of patients in group 1 and 61.54% of those in group 2 developed an aortic complication (*p* < .001). The VIRTUE study (14) found a similar rate of aortic reintervention for patients treated in the acute (20%) and subacute (22%) phases. Qin et al. (19) found 23.90% and 38.30% aortic complications in the TEVAR and OMT groups, respectively. The INSTEAD study (4) found 27% and 46.1% aortic events at 5 years among patients treated with TEVAR and OMT, respectively (*p* = .040). The higher prevalence of aortic events in our OMT group may be explained by the selection of patients with poor prognosis criteria.

The majority of (re)interventions during follow-up were performed for aneurysmal progression, with 16.66% of patients in group 1 and 57.69% in group 2 undergoing surgery for this indication (*p* < .001). Qin et al. (16) found a similar rate with 47.5% aneurysmal evolution. Durham et al. (9) found a 21.7% rate of aneurysmal evolution, with a more heterogeneous population, namely, older, and therefore less at risk of aneurysmal progression.

Late interventions for chronic aneurysmal evolution are often more complex and associated with incomplete anatomic results.

The main event was aneurysmal progression: 8.33% and 53.85% had progression of the thoracic aorta at the last follow-up (*p* < .001), and 12.50% and 76.92% of patients in groups 1 and 2, respectively, had aneurysmal progression of the maximum aortic diameter (*p* < .001). The results in the literature are disparate: Brunkwall et al. (8) found 37% and 45% aneurysmal evolution, whereas Nienaber et al. (4) found 4.1% and 28.1% aneurysmal evolution for patients treated with TEVAR and OMT, respectively. These results can be explained by the fact that we defined aneurysmal progression as an increase in total diameter of >5 mm or a diameter reaching 50 mm in any patient, whereas Brunkwall et al. (8) defined it as an increase of >5 mm or a diameter >55 mm. Furthermore, the INSTEAD trial (4) included all patients managed for uncomplicated TBAD, with or without poor prognosis factors, and in chronic dissections.

It is well known that aortic remodeling after TEVAR is optimal in the subacute phase, with complete healing of the TA aorta, whereas it is suboptimal in the chronic phase. At best, thrombosis of the FL along the stent graft is obtained, without positive remodeling (14, 16). At the end of follow-up, complete aortic remodeling was achieved in 91.67% of patients in group 1 and 42.31% in group 2 (*p* < .001), although 60% of patients in this group underwent surgery during follow-up. The INSTEAD study (4) found 79.2% and 10% complete aortic remodeling at 5 years among chronic patients treated with TEVAR and OMT, respectively. These results confirm the value of early treatment (20).

The favorable anatomical result of early treatment appears to be also linked to the use of the STABILISE technique in most of our patients.

Indeed, the subgroup analysis in group 1 showed that preemptive treatment with TEVAR alone was associated with poorer anatomical results than with preemptive TEVAR–STABILISE. We found significantly less aneurysmal progression (*p* < .010) of the thoracic aorta and stable TA aortic diameters in the STABILISE group (*p* = .090).

In INSTEAD and ADSORB (4, 8), the rate of aneurysmal progression in the preemptive treatment group was 27% at 5 years and 37% at 1 year, confirming the poor results of preemptive TEVAR alone in the treatment of aortic dissections. Solutions other than STABILISE may be considered to ensure optimal aortic remodeling: the Knickerbocker (21), the candy plug (22), or the embolization of the false channel (23).

There was no significant difference in terms of perioperative events (*p* = 1.00). This confirms the interest in selecting at-risk patients who could benefit from such an early intervention without initial excess mortality. In the INSTEAD study (4), the 1-year high mortality rate after TEVAR (7.5% at 1 year) could be explained by the patient inclusion period between 2002 and 2005. Progress in anesthesia, intensive care, stent grafts and endovascular equipment, and a better understanding of the pathology have reduced this mortality, with rates dropping to 0%–5% in recent studies (14, 24, 25).

Several studies have shown better long-term survival in operated patients (14, 24). However, we were unable to show any difference in long-term mortality (*p* = 1.00). This is partly explained by the limited follow-up time, and since it was shown that close, regular monitoring in high-volume referral centers enables early detection of aneurysmal evolution and timely intervention of patients.

## Limitations

Our study must be interpreted with its limitations, namely, its retrospective nature, the low prevalence of the disease, and our restricted inclusion criteria. The heterogeneity among the included patients (acute and subacute phase), could be a bias. The inclusion criteria were very strict, explaining the low number of patients included in our study despite an inclusion period of 11 years. The study aimed to analyze the effect of preemptive treatment (in particular in aortic remodeling) on patients who did not have a formal indication for surgery. However, larger inclusion criteria may have given this study greater power.

Further randomized studies, with a larger number of patients and with an objective endpoint such as all-cause mortality, are needed to confirm the benefit of the preemptive intervention in this at-risk population.

## Conclusion

Preemptive treatment within 30 days for uncomplicated TBAD with poor prognosis factors reduces the risk of long-term aortic events and provides improved complication-free survival and better aortic remodeling. Over 60% of medically treated patients will require intervention during follow-up, most often for aneurysmal progression, with no benefit in terms of early postoperative events or non-aortic complications. Finally, the good results associated with preemptive endovascular treatment seem to be linked to the use of innovative techniques such as STABILISE.

## Data Availability

The raw data supporting the conclusions of this article will be made available by the authors, without undue reservation.
